# Development of a Deep Learning-Based Epiglottis Obstruction Ratio Calculation System

**DOI:** 10.3390/s23187669

**Published:** 2023-09-05

**Authors:** Hsing-Hao Su, Chuan-Pin Lu

**Affiliations:** 1 Department of Otorhinolaryngology-Head and Neck Surgery, Kaohsiung Veterans General Hospital, Kaohsiung 81362, Taiwan; shsu@vghks.gov.tw; 2 Department of Physical Therapy, Shu-Zen Junior College of Medicine and Management, Kaohsiung 82144, Taiwan; 3 Department of Pharmacy and Master Program, College of Pharmacy & Health Care, Tajen University, Pingtung 90741, Taiwan; 4 Department of Information and Communication Engineering, Chaoyang University of Technology, Taichung 41349, Taiwan

**Keywords:** obstructive sleep apnea, epiglottis obstruction, deep learning, computer vision, region puzzle algorithm, drug-induced sleep endoscopy

## Abstract

Surgeons determine the treatment method for patients with epiglottis obstruction based on its severity, often by estimating the obstruction severity (using three obstruction degrees) from the examination of drug-induced sleep endoscopy images. However, the use of obstruction degrees is inadequate and fails to correspond to changes in respiratory airflow. Current artificial intelligence image technologies can effectively address this issue. To enhance the accuracy of epiglottis obstruction assessment and replace obstruction degrees with obstruction ratios, this study developed a computer vision system with a deep learning-based method for calculating epiglottis obstruction ratios. The system employs a convolutional neural network, the YOLOv4 model, for epiglottis cartilage localization, a color quantization method to transform pixels into regions, and a region puzzle algorithm to calculate the range of a patient’s epiglottis airway. This information is then utilized to compute the obstruction ratio of the patient’s epiglottis site. Additionally, this system integrates web-based and PC-based programming technologies to realize its functionalities. Through experimental validation, this system was found to autonomously calculate obstruction ratios with a precision of 0.1% (ranging from 0% to 100%). It presents epiglottis obstruction levels as continuous data, providing crucial diagnostic insight for surgeons to assess the severity of epiglottis obstruction in patients.

## 1. Introduction

According to existing research, 2 out of 10 individuals in Taiwan [1] may be suffering from obstructive sleep apnea (OSA) without knowing it, and at least 800,000 people may be suffering from sleep apnea. OSA symptoms include excessive daytime sleepiness, fatigue, and even lethargy [2]. This condition can also lead to depression in the long run and increase the risk and severity of chronic diseases [3]. To check a patient’s condition and provide the best treatment, it is necessary to know the level and severity of the collapse/obstruction of the patient’s upper airway. Surgeons use endoscopy to examine the soft palate in the supra-glottic area, observe the position of the collapsed soft tissue around the respiratory tract, roughly judge the severity of the collapse, and determine the surgical correction needed for their patient’s condition and the degree of correction. This method is known as drug-induced sleep endoscopy (DISE) [4].

The causes of OSA symptoms are intermittent relaxation of patients’ throat muscles, tonsil hypertrophy, redundancy of the soft palate and uvula, anterior-to-posterior collapse of the tongue base, and epiglottic collapse [5,6,7]. These factors can cause airway obstruction during deep sleep. Common treatments for OSA include continuous positive airway pressure, oral appliance therapy, behavioral modification, and surgical procedures, among others [8]. The treatment method is determined based on the type and degree of obstruction in the affected site. For mild cases, non-invasive treatments should be the first-line management, while invasive surgical treatments are the primary method for severe cases [8,9,10].

The severity of OSA is represented by the apnea–hypopnea index [11], which is defined by the number of apnea and hypopnea events per hour of sleep. Hypopnea refers to a partial airway blockage (a reduction of ≥30% in airflow that lasts for more than 10 s) where blood oxygen saturation or sleep arousal decreases by at least 3%. The current commonly used three obstruction degrees [12] (0–49% collapse (degree 0: no obstruction), 50–75% collapse (degree 1: partial obstruction), and 76–100% collapse (degree 2: complete obstruction)) do not intuitively correspond to the reduction in airflow. Moreover, the difference between 49% “no obstruction” and 50% “partial obstruction” is not significant (the same applies to 75% “partial obstruction” and 76% “complete obstruction”). For instance, if the actual collapse values are “30%, 49%, 50%, 75%, 76%, 100%”, then it would become “0, 0, 1, 1, 2, 2” when represented by the three obstruction degrees. Additionally, if the actual collapses are “50%, 60%, 70%, 75%”, then the obstruction degrees would all be “1,1,1,1”. Collapse values (obstruction ratios) can directly correspond to possible airflow changes, but obstruction degrees are not easily mapped precisely to airflow changes.

Medical information systems based on artificial intelligence have been extensively applied in the medical field in recent years. In situations such as the COVID-19 pandemic, AI healthcare systems can generate multidimensional strategies according to the limited pharmacological resources available to control and minimize casualties from major outbreaks [13]. This is because AI algorithms can generate specific policies based on the importance assigned to casualties or different policies in each sub-model. In recent years, deep learning has been regarded as a significant benchmark in the field of artificial intelligence. It has achieved remarkable results in various complex cognitive tasks, particularly surpassing human performance in tasks such as image matching. Deep learning neural network algorithms that were developed based on the core architecture of convolutional neural networks (CNNs) [14,15] include AlexNet [16], GoogLeNet [17], SSD [18], and YOLOv1–v3 [19,20,21], as well as YOLOv4 and v7 [22,23,24]. CNNs offer a non-parametric approach to prediction and recognition. Recently, advanced parametric, multi-dimensional, constrained, and probabilistic machine learning algorithms have been developed to provide long-term, stable, and optimal predictions and strategic formulations. Currently, common programming languages that can implement CNNs include C++ and Python, with corresponding development tools such as Microsoft Visual Studio, Anaconda, and Jupyter Notebooks. EKEN introduced medical data analysis notebooks for collaborative and reproducible research across different data types in a 2020 study [25]. This information is immensely beneficial for scholars who are just starting to explore deep learning. Numerous studies on the application of information technology in OSA detection have been conducted. For example, Ferrer-Lluis et al. used smartphones and PSG signals to monitor patients’ sleep status and found a correlation between sleep posture and OSA [26]. Kou et al. proposed a deep learning-based unsupervised model that was applied to esophageal manometry to learn the distinctive patterns of clinical manifestations associated with esophageal motility disorders [27]. In 2022, Liu et al. applied snoring sounds to classify obstruction sites in OSA [28]. Snoring sounds were recorded during the DISE examination of OSA patients. Hanif et al. used a deep learning approach for the automatic scoring of drug-induced sleep endoscopy in obstructive sleep apnea [29]. In their study, the research objective was to identify the degree of obstruction in the diseased sites (velum, oropharynx, tongue base, and epiglottis). The obstruction levels were categorized into three degrees. ResNet18 was used for feature extraction of DISE video frames, and all feature vectors were concatenated and input into a bidirectional long short-term memory network for temporal analysis. These neural networks were then used to recognize the three degrees of obstruction for each diseased site [12].

Evaluating the degree of obstruction in an affected area manually using DISE videos is not only inefficient but also lacks precision. Therefore, Hanif et al.’s study [29] utilized deep learning to automatically score the degree of OSA obstruction. Their method was based on the grading criteria introduced by Kezirian in 2011 [12], using images of three degrees of OSA obstruction as the target for deep-learning image training. This research treated images showing the three degrees of obstruction from DISE videos as three classes for training the neural network. The experimental results indicated that the average recognition rate (F1 score) for epiglottic obstruction was 65%. However, when looking only at the presence or absence of obstruction, the accuracy increased to 91%. Upon observing the confusion matrix, the recognition effectiveness for the three obstruction degrees was not good. The representation of the three obstruction degrees was neither precise nor intuitive. Compared to obstruction degrees, an obstruction ratio can offer surgeons a more accurate diagnosis. To expand the calculation of the obstruction ratio from the three degrees of obstruction to a numerical ratio, this study proposed a new method for analyzing epiglottic obstruction images to calculate the obstruction ratio and integrate it into a web system for ease of use. The method proposed in this research can intuitively and accurately calculate the obstruction ratio of an epiglottic region (ranging from 0% to 100%).

This research developed a computer vision system for the automatic calculation of the epiglottic obstruction ratio. This system utilizes a client–server network architecture and integrates both web-based and PC-based technologies, thus enabling cross-platform usability. The image analysis method used in this study involves utilizing video frames recorded during endoscopic inspections to detect the range of the airway (termed field of view (FOV); the larger the FOV, the lower the obstruction ratio), presenting the airway range data as continuous values, and calculating the time and proportion of epiglottic obstruction from different endoscopic views. The detection method for airway range uses the YOLOv4 model to locate the epiglottic cartilage in an image and then employs the median-cut color quantization method [30] to transform the image into color regions. Subsequently, the proposed region puzzle algorithm (RPA) is used to obtain the image regions of the epiglottic cartilage and the airway range (FOV). The RPA is a region-merging algorithm based on region location and geometric shape. The merging of regions is performed by taking the color regions as the basic units, and the airway region is obtained through the merging process. Finally, the obstruction ratio is calculated based on the continuous data of the FOV and the location of the endoscopy in the throat. The algorithm’s effectiveness and practicality were verified by comparing its results with clinical judgment results. The detailed structure and process of this system are introduced in the following sections. 

## 2. Methods

### 2.1. System Architecture and Processing Procedures

**Figure 1:**
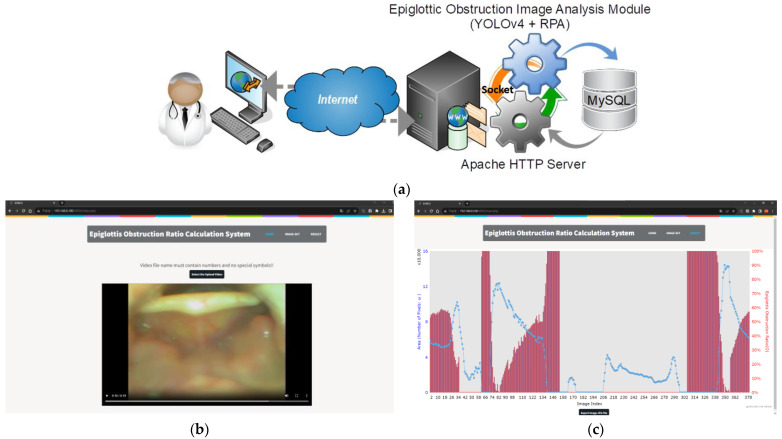
The proposed system: (**a**) architecture; (**b**) the user interface of the web page for the upload of DISE videos; and (**c**) web page showing the generated chart with epiglottis obstruction ratios.

**Figure 2:**
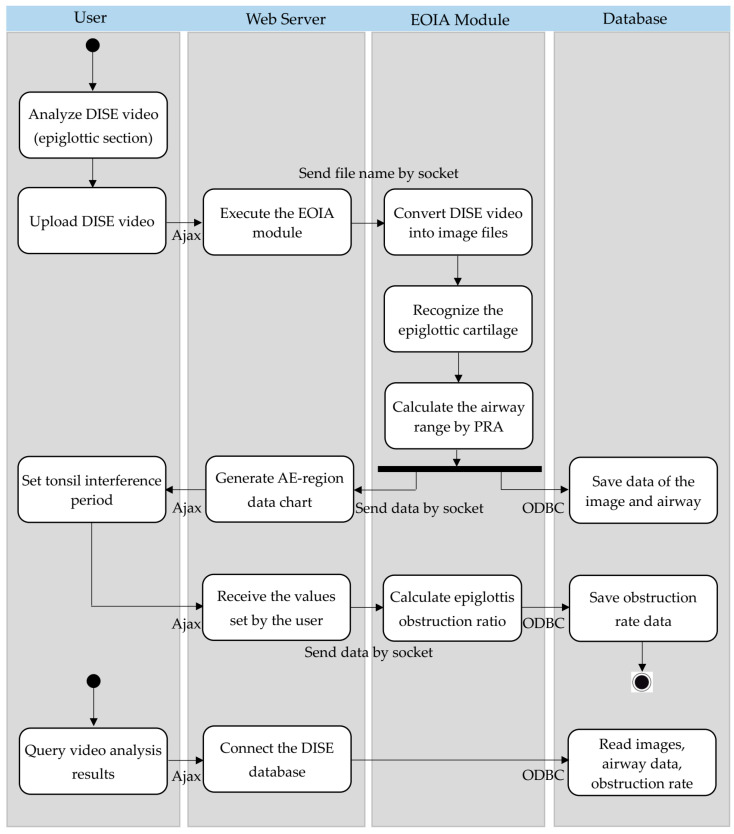
The flowchart (activity diagram) of the proposed system.

The proposed system adopts a client–server architecture and uses a web platform to provide remote access for users, thus offering the convenience of cross-platform compatibility (see Figure 1a). The front-end web interface was developed using HTML5 (see Figure 1b,c), while the back-end website system was developed to operate on Apache HTTP Server, utilizing PHP programming to implement the system workflow (including upload of endoscopic videos, transmission of socket messages to the Image Analysis Module, generation of an epiglottis obstruction rate chart, and report generation). Figure 1b shows the web interface that allows users to upload DISE videos, while Figure 1c shows the obstruction rate and the visible airway area curve obtained after the system processes the DISE videos. After a system user completes the upload of endoscopy videos and initiates the image analysis execution, the Apache Server sends a message to the Image Analysis Module (PC-based) via a socket. The Epiglottic Obstruction Image Analysis (EOIA) Module reads the video files in the directory, conducts frame capturing from the videos, and carries out the YOLOv4, RPA, and other required image processing algorithms. The execution results are stored in a MySQL database. A socket message is then sent back to the Apache Server, notifying it that the image analysis task is completed. In addition, this system uses Asynchronous JavaScript and XML (Ajax) for front-end and back-end communication. Both the Apache Server and EOIA connect to the MySQL database using Open Database Connectivity (ODBC). The system’s flowchart (activity diagram) is shown in Figure 2.

### 2.2. FOV Detection of Epiglottis Airway

When patients with OSA, who have epiglottic collapse, fall asleep, the epiglottis is impelled to shift against the posterior pharyngeal wall by the negative pressure airflow during inspiration, and obstruction happens. In the case that the endoscopy is placed in front of the epiglottis and the airway is not completely obstructed, the obtained endoscopy image shows the arytenoid cartilage, aryepiglottic folds, hypopharynx, and vocal cords. This is called the airway’s FOV. It may show the patient’s lax epiglottis collapse at the entrance of the larynx or the tissues above the epiglottis collapse (especially the palatine tonsils on both sides) [31]. In such a case, the epiglottis collapse narrows or completely obstructs the airflow channel, thus reducing the airway’s FOV. Based on the above phenomenon, the proposed research method aims to find the FOV of the airway. For ease of explanation, we call the FOV of the airway the “AE-region”. The endoscopy, which is placed in front of a patient’s epiglottis cartilage, shows the epiglottis, arytenoid, aryepiglottic fold, vocal fold, and glottis. In addition, it shows the posterior and lateral pharyngeal walls. The AE-region must exclude the epiglottis and the site of the posterior and lateral pharyngeal walls. Therefore, the proposed RPA is used to analyze the AE-region from an image and use it as the range of the airflow path.

**Figure 3:**
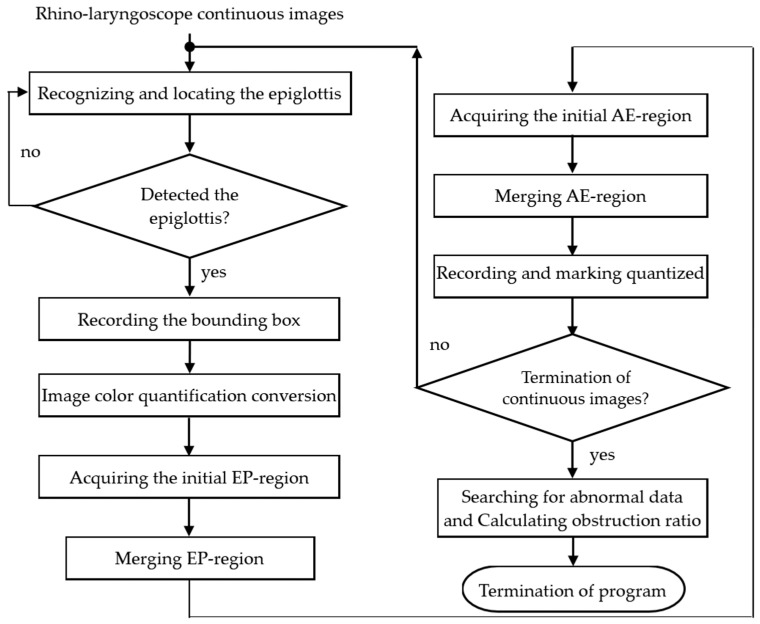
The process of AE-region detection.

We use the YOLOv4 model to locate the epiglottis for two main reasons. The first reason is to mark the images of the endoscopy’s FOV and acquire the maximum AE-region of the period as a reference value for the unobstructed airway range. The image where the epiglottic cartilage is recognized for the first time is regarded as the starting point of the new period, and the period number m is set to 1. The first image where the epiglottic cartilage cannot be recognized indicates that the FOV of the endoscopy has changed and is regarded as the last image of the period. When the epiglottis is recognized again, the period number is updated to m+1, and another period begins. The program repeats the detection of the start and end points repeatedly. The second reason is to find the AE-region of an epiglottis image. The bounding box of the epiglottis can be obtained using the YOLOv4 model. Then, the 24-bit image is converted into color regions using the median-cut color quantization method [30], and all regions are subsequently labeled [32]. The region located in the center of the bounding box is used as the initial region of the epiglottis. Within the bounding box range, we use the RPA to merge the surrounding adjacent regions one by one. After merging several times, the epiglottis region (EP-region) is obtained. Then, we use the coordinates at a specific point in the EP-region as the basis for finding the starting region of the AE-region, and we execute the RPA to obtain the AE-region of each image. If an image detects the epiglottis and not the AE-region, then it indicates that the epiglottis is completely obstructed. Simultaneously, we draw the AE-region area of the continuous image into a curve, search for abnormal values in the data, and mark these values (not counting the obstruction ratio). There are two ways to determine whether a value is abnormal. The first is that the detection result of the front image and the back image does not contain the epiglottis or the epiglottis is completely obstructed but the current image presents a different result. Then, the detection result of the image is regarded as abnormal. The second way is to use backward difference calculation and compare the before and after values of the data group with the threshold value. If the data group meets the conditions, the value that is higher than the threshold value (ε) is regarded as abnormal. Finally, the largest AE-region in each period is used as the denominator to calculate the obstruction ratio of the period. The calculation of the obstruction ratio ($O_{m}^{n}$) (percentage) is shown in Equation (1), where A denotes the AE-region, $\left|A\right|$ denotes the number of elements in the set (number of pixels), and n is the image index of continuous frames. The process of AE-region detection is shown in Figure 3 (the endoscopy used in this study is a rhino-laryngoscope):

(1) $$O_{m}^{n}(\%)=100\times (\max\left\{{|A}_{m}^{n}|\right\}-{|A}_{m}^{n}|)/max\{{|A}_{m}^{n}|\},$$

### 2.3. You Only Look Once Version 4 Model

The advantages of the YOLO model for object positioning have been confirmed in previous research [33,34,35]. Bochkovskiy, Wang, and MarkLiao innovatively introduced YOLOv4 in April 2020 [22]. To further enhance the performance of YOLOv4, they also proposed a strategy known as network scaling [23]. Compared to YOLOv3, significant improvements were made to the architecture of YOLOv4. Firstly, in the backbone part of the original Darknet53 network, the authors incorporated Cross Stage Partial Connections (CSP), which effectively reduced the network parameters and computational load while maintaining excellent accuracy. This new architecture is called CSPDarknet53 [36]. Secondly, in the neck part of the network, the original Feature Pyramid Network was replaced by the PANet (Path Aggregation Network). The PANet aggregates the backbone parameters corresponding to detectors at different levels. This section also employs the Spatial Pyramid Pooling strategy, which strengthens the effect of the receptive field by separating the context. In the head part used for predicting object classes and drawing bounding boxes, the one-stage Dense Prediction strategy of YOLOv3 is retained. In addition, YOLOv4 introduces a non-monotonic and continuously differentiable Mish activation function $M\left(x\right)$ [37] (see Equation (2)), which allows gradients to be more effectively propagated without causing a significant increase in computational cost:

(2) $$M\left(x\right)=xtanh\left(\ln\left(1+e^{x}\right)\right)$$

For the bounding box regression loss part, YOLOv4 uses the Complete IoU (CIoU) Loss to replace the original mean squared error loss. The function of the IoU Loss is to calculate bounding box coordinate regression, source prediction, and class score prediction. The CIoU Loss (φ) is formulated as follows [38]:

(3) $$\varphi =1-IoU+\Psi ,\;\;\;\;\;\Psi =\frac{\vartheta^{2}\left(ed,{ed}^{t}\right)}{c^{2}}+\alpha v,$$

(4) $$\alpha =\frac{4}{\pi^{2}}{\left(\tan^{-1}(\frac{{bw}^{t}}{{bh}^{t}})-\tan^{-1}(\frac{bw}{bh})\right)}^{2},$$

(5) $$v=\frac{\alpha }{\left(1-IoU\right)+\alpha },$$

where the parameter Ψ is the penalty term for the predicted box and the target box; $\vartheta^{2}\left(ed,ed^{t}\right)$ is the Euclidean distance between the predicted box and the target box; c is the diagonal length of the smallest enclosing box covering the two boxes; α evaluates the consistency in the aspect ratio between the predicted bounding box and the ground truth; and v serves as a trade-off parameter to balance the scale. α and v are calculated using Equation (4) and Equation (5), respectively. In Equation (4), ${bw}^{t}$ and ${bh}^{t}$ denote the width and height of the target box, while bw and bh denote the width and height of the predicted box. When the differences in width, height, and distance between the predicted box and the target box are significant, this penalty term will increase the loss value; otherwise, it will not affect the loss value.

In terms of data augmentation strategies, YOLOv4 adopts the Mosaic method aiming at reducing GPU (Graphics Processing Unit) computational load and memory requirements. During the training process, a DropBlock strategy is used to prevent the model from overfitting, and a Modified Spatial Attention Module method is used to further enhance the network’s representation power. This method applies YOLOv4 to locate the epiglottis due to its fast calculation speed, good mAP, and good identification results. The C++ version currently only supports YOLOv4, while YOLOv7 is supported in the Python (Pytorch Framework) version. As this research aims at practical application in hospitals, YOLOv4 was chosen as the object detection model for the system.

### 2.4. Region Puzzle Algorithm

The concept of this algorithm is similar to a jigsaw puzzle. The edges of the puzzle board are more curved at the beginning, but as the puzzle gets closer to completion, the edges smooth out. The RPA is used based on the concept of puzzles, and the setting with a low color quantity quantifies the image setting. The converted color region (denoted $R_{j}$) is combined within the analysis region and merged into the target region (denoted T, the EP- and AE-regions). $R_{j}$ is known as the “puzzle pieces.” In the kth merge procedure (k≥1), we check whether $R_{j}$ fulfills two conditions around $T^{\left(k-1\right)}$: (1) a single region $R_{j}$ is completely adjacent to the surrounding region $T^{\left(k-1\right)}$, and (2) $R_{j}$ does not exceed the scope of the analysis region. If both conditions are met, then $T^{\left(k-1\right)}$ will merge with $R_{j}$ to form $T^{\left(k\right)}$. To speed up the merging operation, regions with holes are filled after each merge. In the k+1 merge, the puzzle pieces $R_{j}$ are based on the total number of points on edge after region merging (defined as the puzzle function $P_{j}^{\left(k+1\right)}$). This is used to determine whether $R_{j}$ should include $T^{\left(k\right)}$. This algorithm calculates the edge line $C_{j}^{\left(k+1\right)}\left(=Edge\left(R_{j}\cup T^{\left(k\right)}\right)\right)$ of the region when merging $R_{j}$ with $T^{\left(k\right)}$. The kth merge of the puzzle function $P^{\left(k\right)}$ is defined as shown in Equation (6). The k+1th merge puzzle function $P_{j}^{\left(k+1\right)}$ is defined as shown in Equation (7); the edge of $T^{\left(k\right)}$ is defined as $C^{\left(k\right)}$, with $C^{\left(k\right)}$ being obtained through the gradient operation processing [14] of $T^{\left(k\right)}$, and $\omega_{j}$ is the weight value of $R_{j}$. The algorithm performs the exclusive-OR (XOR) operation on the edge $D_{j}$ of $R_{j}$ and the edge $C^{\left(k\right)}$ of $T^{\left(k\right)}$. The XOR operation can subtract the overlapped part of $D_{j}$ and $C^{\left(k\right)}$. The result of the operation is $P_{j}^{\left(k+1\right)}$. If $P_{j}^{\left(k+1\right)}>P^{\left(k\right)}$, then $R_{j}$ is not merged with $T^{\left(k\right)}$; if $P_{j}^{\left(k+1\right)}\leq P^{\left(k\right)}$, then $R_{j}$ is merged with $T^{\left(k\right)}$, and the result is updated to $T^{\left(k+1\right)}$, $C^{\left(k+1\right)}$, and $P^{\left(k+1\right)}$. The merging process is repeated until $P^{\left(k+i\right)}=P^{\left(k+i+1\right)}$, when the best result of the puzzle of the target region T is acquired.

The puzzle function for the kth iteration:

(6) $$P^{\left(k\right)}=\left|C^{\left(k\right)}\right|$$

The puzzle function for the k+1th iteration:

(7) $$P_{j}^{\left(k+1\right)}=\left|C_{j}^{\left(k+1\right)}\right|-w_{j}=\left|\left(D_{j}⊕C^{\left(k\right)}\right)\right|-w_{j},w_{j}=Y_{j}-Y_{e}/2$$

**Figure 4:**
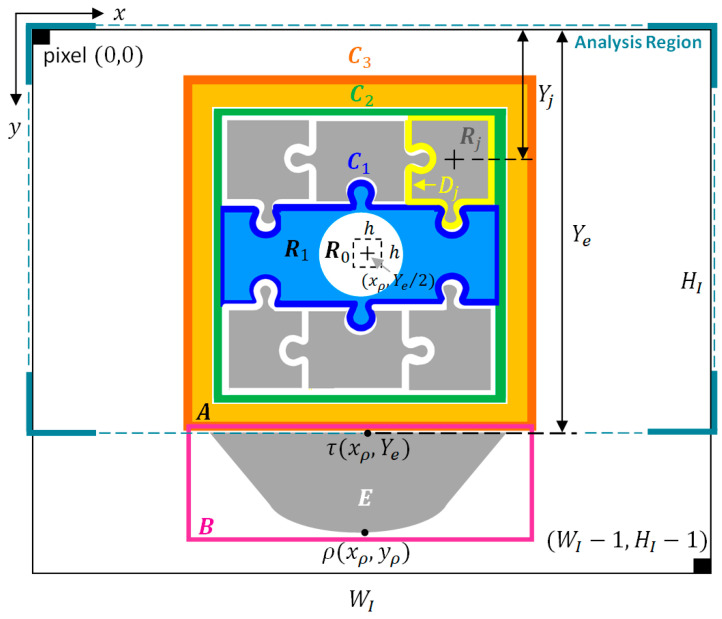
A diagram of AE-region merging using RPA.

Among them, $P^{\left(k\right)}$ is the puzzle function for the kth iteration of $T^{\left(k\right)}$, $P_{j}^{\left(k+1\right)}$ is the puzzle function under the k+1th execution of the program, and when $R_{j}$ and $T^{\left(k\right)}$ are merged, $\left|C\right|$ denotes the number of elements in the set C, while $w_{j}$ is the weight of $R_{j}$. The closer $R_{j}$ is to the EP-region, the bigger the value of $w_{j}$, and the further away $R_{j}$ is from the EP-region, the smaller the value of $w_{j}$. $Y_{j}$ is the vertical distance from the $R_{j}$ region. $Y_{e}$ is the vertical distance from the edge point $\tau \left(x_{\rho },Y_{e}\right)$ on the EP-region (Figure 4). In Figure 4, A represents the AE-region; B indicates the Bounding Box’s region; $C_{1}$, $C_{2}$, and $C_{3}$ are the edge lines of the puzzle pieces.

The following shows how the RPA merges the EP-region and the AE-region in practice. There are two differences between the two regions: one is the selection of the initial region and the other is the analysis region range.

#### 2.4.1. EP-Region Merging

**Figure 5:**
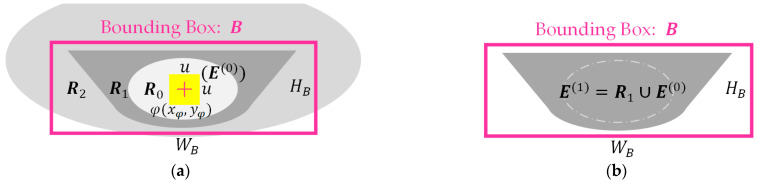
A diagram of EP-region merging: (**a**) the initial region $E^{\left(0\right)}\left(=R_{0}\right)$ and (**b**) the final merged result $E^{\left(1\right)}\left(=R_{1}\cup E^{\left(0\right)}\right)$.

After the YOLOv4 model finds the bounding box (denoted B) of the epiglottis, we start to merge the regions defined by B, thereby yielding the target region (EP-region, denoted E). First, in the u×u range (center of $\varphi \left(x_{\varphi },y_{\varphi }\right)$), we find the brightest region $R_{0}$ (with the largest RGB color value, such as $R_{0},$ as shown in Figure 5) as the puzzle board (the initial region $E^{\left(0\right)}$ of the epiglottic cartilage, k=0). Then, we find the surrounding regions of $R_{0}$ (such as $R_{l}$, with l=1,2,3,…), and merge $E^{\left(0\right)}$ with $R_{l}$ repeatedly. If $R_{l}$ exceeds the range of B, it will not be merged, and the result of the $E^{\left(k+i\right)}$ merge will be defined as $E^{\left(1\right)}$. The above steps are repeated. If $P^{\left(k+i\right)}$ is the same as $P^{\left(k+i+1\right)}$, merging will be stopped, and $E^{\left(k+i\right)}$ is the merged result of the EP-region. As shown in Figure 5 as an example, $H_{B}$ is the height of B, and $W_{B}$ is the width of B. In Figure 5a, $E^{\left(0\right)}$ is the initial region of the epiglottis, and *u* is set to a value between $H_{B}/4$ and $H_{B}/2$, which means that the surrounding region $R_{1}$ of the initial region $E^{\left(0\right)}$ does not exceed the analysis range B, and is completely adjacent to the surrounding region $E^{\left(0\right)}$. The range of $R_{2}$ that exceeds B will not be merged; therefore, $E^{\left(1\right)}\left(=R_{1}\cup E^{\left(0\right)}\right)$ is the final merged result (Figure 5b). The common EP-region is similar to the type shown in Figure 5. In addition, if the range of $R_{0}$ exceeds the bounding box, we use it as the epiglottis region and stop the merging procedure.

#### 2.4.2. AE-Region Merging

The RPA merges the $R_{j}$ in the analysis region to obtain the target region (AE-region, denoted A). We demonstrate this in Figure 4. The common AE-region is similar to Figure 4. $H_{I}$ and $W_{I}$ in Figure 4 are the height and width of the image, respectively, and the pixel $\left(0,0\right)$ is the origin of the image, while $\left(W_{I}-1,H_{I}-1\right)$ is the last point of the image. Before performing this algorithm, we first find the highest point $\rho \left(x_{\rho },y_{\rho }\right)$ and the edge point $\tau \left(x_{\rho },Y_{e}\right)$ of the EP-region E. The analysis region is the range within $Y_{e}$ corresponding to the h×h range at $\left(x_{\rho },Y_{e}/2\right)$ everywhere (h is between $Y_{e}/4$ and $Y_{e}/2$). Next, we find the darkest color in this range $R_{0}$ (where the RGB color value is the smallest) and use it as a puzzle board and as the initial region $A^{\left(0\right)}$ of the AE-region ($A^{\left(k\right)}$, k=0). $A^{\left(k\right)}$ is the AE-region after the kth merge. After detecting $R_{0}$, we set it to $A^{\left(0\right)}$ and execute the RPA for region-merging analysis. The algorithm analyzes the puzzle functions of the kth merging process of $R_{j}$ and $A^{\left(k-1\right)}$ one by one with the puzzle function $P_{j}^{\left(k\right)}\left(=\left|C_{j}^{\left(k\right)}\right|-w_{j}\right)$. The merging process is repeated until $P^{\left(k+i\right)}=P^{\left(k+i+1\right)}$, upon which the program is stopped, outputting the best result of the AE-region A. As shown in Figure 4, we describe this in a sequence of steps for AE-region merging using the RPA:

Step 1. Find the region covered by the h×h range at the location $\left(x_{\rho },Y_{\tau }/2\right)$ as the initial region $A^{\left(0\right)}$ and fill any holes within $A^{\left(0\right)}$.
Step 2. In the kth merging procedure (k≥1), check whether the region surrounding $A^{\left(k-1\right)}$ and region $R_{j}$ meets two conditions: (1) $A^{\left(k-1\right)}$ is surrounded by a single region $R_{j}$, and (2) the range of $R_{j}$ does not exceed $Y_{e}$. If the two conditions are satisfied, $A^{\left(k-1\right)}$ will be directly merged with $R_{j}$ and the AE-region is updated to be $A^{\left(k\right)}$. Fill in the holes in $A^{\left(k\right)}$, and proceed to Step 3.
Step 3. Perform the edge operation processing on $A^{\left(k\right)}$ to obtain the edge line $C^{\left(k\right)}$ and calculate the puzzle function $P^{\left(k\right)}$.
Step 4. Search for puzzle pieces $R_{j}$ adjacent to $A^{\left(k\right)}$, and calculate the edge lines $D_{j}$ of $R_{j}$.
Step 5. Perform an XOR operation on each edge $D_{j}$ of $R_{j}$ with $C^{\left(k\right)}$ and calculate $P_{j}^{\left(k+1\right)}$. If $P_{j}^{\left(k+1\right)}\leq P^{\left(k\right)}$, merge $R_{j}$ with $A^{\left(k\right)}$, and vice versa. After completing all the surrounding analysis of $R_{j}$, fill in the holes of $R_{j}$.
Step 6. Compare the values of $P^{\left(k\right)}$ and $P^{\left(k+1\right)}$. If $P^{\left(k\right)}\leq P^{\left(k+1\right)}$, stop the algorithm. $A^{\left(k\right)}$ is the best result. If $P^{\left(k\right)}>P^{\left(k+1\right)}$, update the AE-region result to be $A^{\left(k+1\right)}$, and repeat steps 2 through 6.

## 3. Results

**Figure 6:**
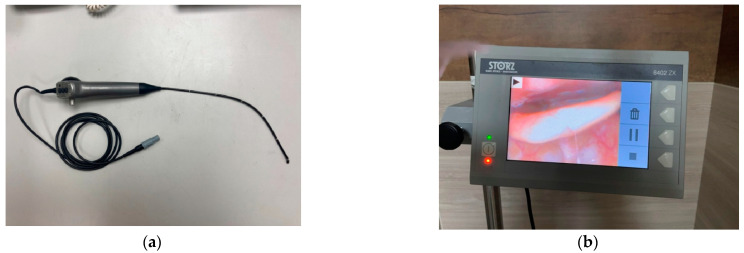
This study’s experimental endoscopy: (**a**) CMOS video rhino-laryngoscope and (**b**) 8402 ZX monitor.

Six experiments were performed to verify the effectiveness of the method proposed in this study, namely, epiglottis image positioning, EP-region merging, AE-region merging, the effect of color quantification on the AE-region, analysis of continuous image AE-region change, and comparison of the proposed method with the clinical judgment results. This study’s experimental equipment and development tools are as follows: Epiglottis images were captured from the epiglottis of OSA patients and recorded during endoscopy (frame rate: 30 frames/s, image resolution: 640 × 480 (pixels)). Endoscopy was performed using a KARL STORZ 3.7 mm CMOS video rhino-laryngoscope (11101CMK was manufactured by KARL STORZ in Tuttlingen, Germany; Figure 6a) connected with an 8402 ZX monitor (8402 ZX monitor was manufactured by KARL STORZ in Tuttlingen, Germany; Figure 6b), and the video recording file format was Mpeg4. The patients underwent the DISE method at the Kaohsiung Veterans General Hospital to evaluate the obstruction sites during sleep. After the patients fell asleep, the surgeon placed the rhino-laryngoscope in front of the epiglottis and recorded the collapse/obstruction of the epiglottis during the inspiration phase. The IRB of the authors’ institution approved this research, with approval no. IRB-KSVGH20-CT2-02. The image analysis algorithm and YOLOv4 implemented in this research were written and executed in the C++ programming language. The computer hardware included an Intel(R) Core (TM) i7 2.8 GHz Central Processing and NVIDIA RTX A4500 GPU graphics card for epiglottis image training and executing the proposed method.

### 3.1. Epiglottis Image Positioning by the YOLOv4

We collected 2230 epiglottis images for training the neural network (1784 images were used for model training (80%), and 446 images were used for model validation (20%)) and labeled the epiglottis images using LabelImg [39]. During the training of the sample, the parameters set by YOLO4 were used in this study. The configuration file was set as follows:

1. The batch size and the mini-batch size are 32 and 8, respectively.
2. The image input to the network is 24-bit color images with 416 × 416 pixels.
3. The activation function is Mish.
4. The regional comparison rate is 0.9 (Momentum = 0.949).
5. The weight reduction ratio is 0.0005 (Decay = 0.0005).
6. The sample image diversification parameters are set to Angle = 0, Saturation = 1.5, Exposure = 1.5, and Hue = 0.1.
7. The network learning rate is 0.001 (Learning Rate = 0.001).
8. The maximum number of iterations is 5000 (Max Batches = 5000).
9. The learning policy is “Step”.
10. The number of feature generation filters is 32 (Filter = 32).
11. The number of object classes is 1 (epiglottis) (Classes = 1).

**Figure 7:**
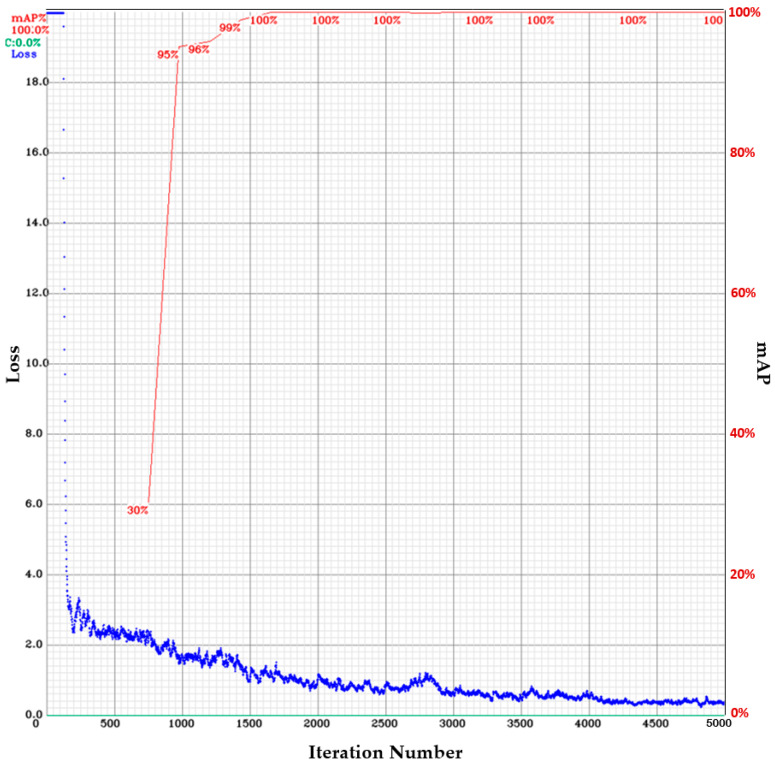
A chart of the loss value and mAP in the epiglottis images trained using YOLOv4 (the blue curve represents the loss value, while the red curve represents the mAP value).

**Figure 8:**
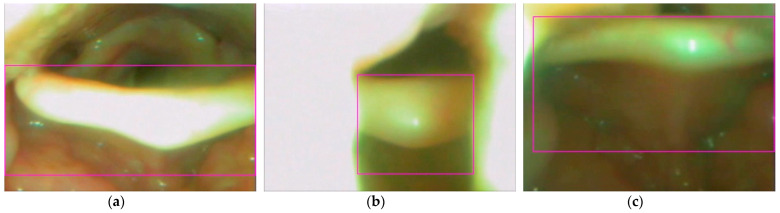
Three epiglottis images’ positioning (shown in bounding boxes of fuchsia color) based on YOLOv4: (**a**) case 1; (**b**) case 2; and (**c**) case 3.

The YOLOv4 model was used to perform 5000 iterations of training of the obtained epiglottis images. A chart of the loss value and mAP during training is shown in Figure 7 (for the training samples: the average loss is 0.3186; for the validation samples: IoU_threashold is set as 0.75, mAP is 87.92%, Precision is 0.91, Recall is 0.91, and F1 score is 0.91). The horizontal axis of Figure 7 represents the iteration number, the left vertical axis indicates the loss value, and the right vertical axis shows the mAP value. For this application, the epiglottis was the only recognized object. A rectangular box (bounding box) is used to mark the epiglottis image. Figure 8 shows the recognized results of three cases.

### 3.2. EP-Region Merging

**Figure 9:**
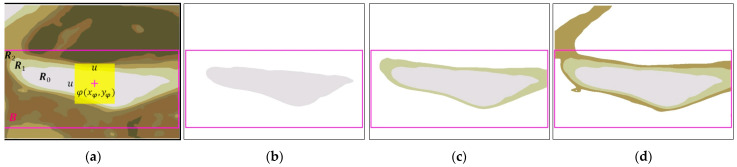
EP-region merging of case 1 ($E^{\left(1\right)}$) within the bounding box: (**a**) color-quantized image; (**b**) $E^{\left(0\right)}\left({=R}_{0}\right)$; (**c**) $E^{\left(1\right)}$; and (**d**) $R_{2}$ exceeds the range B.

**Figure 10:**
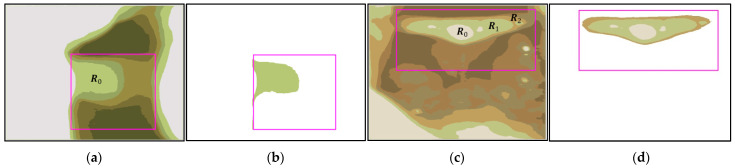
EP-region merging of case 2 and case 3: (**a**) case 2; (**b**) EP-region $E^{\left(0\right)}$; (**c**) case 3; and (**d**) EP-region $E^{\left(2\right)}(=R_{2}\cup E^{\left(1\right)}{{=R}_{2}\cup (R}_{1}\cup E^{\left(0\right)})={R_{2}\cup R}_{1}\cup R_{0})$.

After obtaining each image’s bounding box (B) of the epiglottis, we used the median-cut method to perform color quantization (the number of quantized colors, q=6). The color-quantized image is shown in Figure 9a, and all regions are marked. $\varphi \left(x_{\varphi },y_{\varphi }\right)$ in Figure 9a is the center of the region B, and the h×h range in the experiment is 60×60 pixels. Figure 9a shows the image of Figure 8a case 1 after color quantization. We found and denoted the brightest region in the 60×60 range as $R_{0}$ and used it as the initial region $E^{\left(0\right)}$ (Figure 9b). Then, we detected region $R_{1}$ that is adjacent to the initial region $E^{\left(0\right)}$. $R_{1}$ meets the condition that it is within the range of B, so $R_{1}$ and the initial region $E^{\left(0\right)}$ were merged to become $E^{\left(1\right)}$. As shown in Figure 9c, the program continued to detect region $R_{2}$ that surrounds $E^{\left(1\right)}$. Since $R_{2}$ exceeds the range B (Figure 9d), $E^{\left(1\right)}$ was not merged with $R_{2}$, and the EP-region is $E^{\left(1\right)}$ (${(R}_{1}\cup R_{0})\subseteq B$). Figure 10 shows the EP-region merging results of case 2 and case 3 in Figure 8. Case 2 in Figure 10a is directly used as the EP-region (Figure 10b) because the range of $R_{0}$ exceeds B; Figure 10c, which shows case 3, combines the three regions (${(R_{2}\cup R}_{1}\cup R_{0})\subseteq B$) as the EP-region $E^{\left(2\right)}$, and the result is shown in Figure 10d.

### 3.3. AE-Region Merging

**Figure 11:**
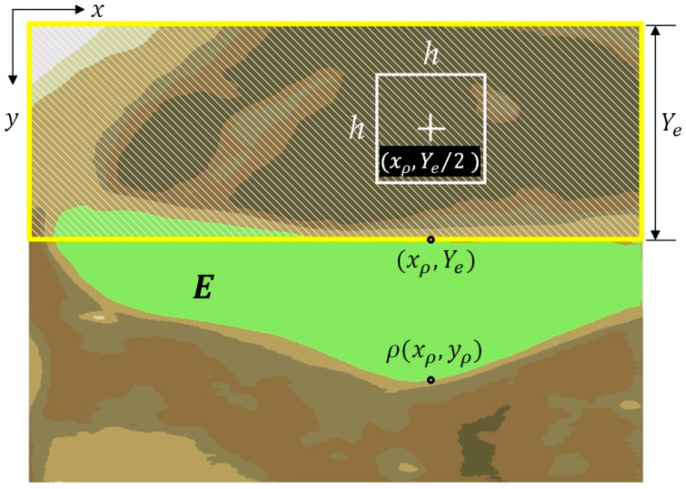
Analysis of the AE-region (the yellow box with diagonal brush style).

**Figure 12:**
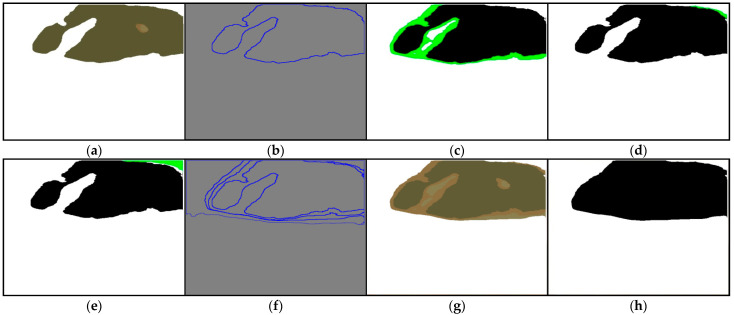
AE-region merging of case 1: (**a**) $A^{\left(0\right)}$; (**b**) $C^{\left(0\right)}$; (**c**) $R_{1}$; (**d**) $R_{2}$; (**e**) $R_{3}$; (**f**) $C^{\left(1\right)}$; (**g**) $R_{2}\cup R_{1}\cup R_{0}$; and (**h**) $A^{\left(1\right)}$.

**Figure 13:**
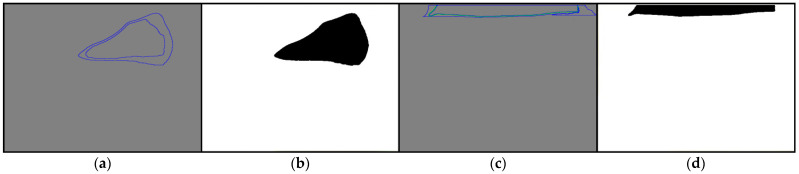
AE-region merging of case 2 and case 3: (**a**) $C^{\left(1\right)}$ of case 2; (**b**) $A^{\left(1\right)}$; (**c**) $C^{\left(0\right)}$ of case 3; and (**d**) $A^{\left(0\right)}$.

As shown in Figure 11, the RPA comprises six steps. In Step 1, we acquired the highest point $\rho \left(418,371\right)$ of the EP-region. Then, we calculated the 56 × 56 $\left(h\times h\right)$ range at coordinate $\left(x_{\rho },Y_{e}/2\right)=\left(418,112\right)$. We chose the region $R_{0}$ with the smallest RGB value and used it as the initial region $A^{\left(0\right)}$ of the AE-region. We then filled in the holes of $A^{\left(0\right)}$ (Figure 12a). In Step 2, we checked whether $A^{\left(0\right)}$ is surrounded by a single region. In Step 3, we used edge calculations [14] on $A^{\left(0\right)}$ to acquire the puzzle function $P^{\left(0\right)}\left(=\left|C^{\left(0\right)}\right|\right)$ with edge line $C^{\left(0\right)}$. In Step 4, we found nearby puzzle pieces $R_{j}=\left\{R_{1},R_{2},R_{3}\right\}$ along $C^{\left(0\right)}$ (Figure 12b). $R_{1}-R_{3}$ are shown in Figure 12c–e, respectively. The edge lines of these regions are $D_{1}$, $D_{2}$, and $D_{3}$, respectively. In Step 5, we performed XOR calculation on $C^{\left(0\right)}$ and $D_{1}$, $D_{2}$, and $D_{3}$. The puzzle function of $C^{\left(0\right)}$ and $D_{1}$ and $C^{\left(0\right)}$ and $D_{2}$ are $P_{1}^{\left(1\right)}\left(<P^{\left(0\right)}\right)$ and $P_{2}^{\left(1\right)}\left(<P^{\left(0\right)}\right)$, so we merged $A^{\left(0\right)}$ with $R_{1}$ and $R_{2}$ to acquire $A^{\left(1\right)}{=(R_{2}\cup R}_{1}\cup A^{\left(0\right)})$. The puzzle function of $C^{\left(0\right)}$ and $D_{3}$ is $P_{3}^{\left(1\right)}$, and as $P_{3}^{\left(1\right)}{>P}_{0}$, no merging was performed. In Step 6, we updated the puzzle function to be $P^{\left(1\right)}$ and the AE-region to be $A^{\left(1\right)}$, and repeated steps 2 through 6. Figure 12f shows the edge line $D_{j}$, Figure 12g shows the merging of $R_{1}$ and $R_{2}$ with $R_{0}$. Figure 12h shows $A^{\left(1\right)}$ (the best result). Figure 13 shows the analyzed results of the AE-regions of case 2 and case 3 in Figure 8. These three cases are common forms of AE-regions.

### 3.4. The Effect of Color Quantification on the AE-Region

**Figure 14:**
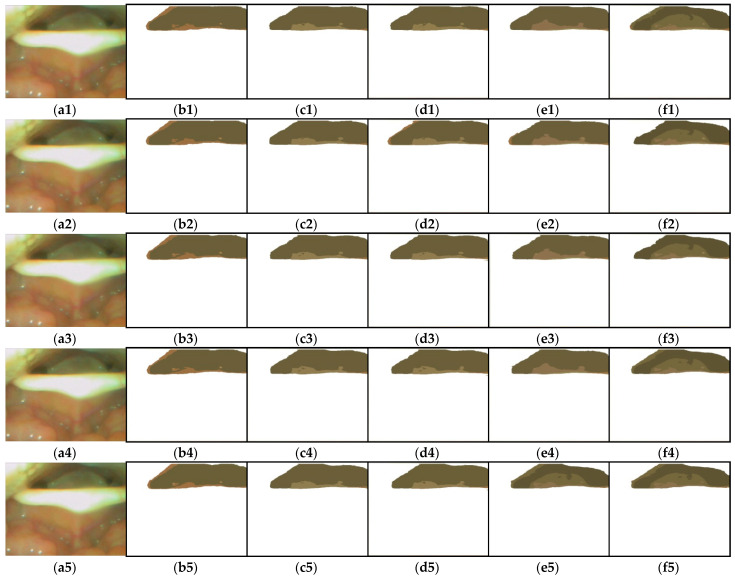

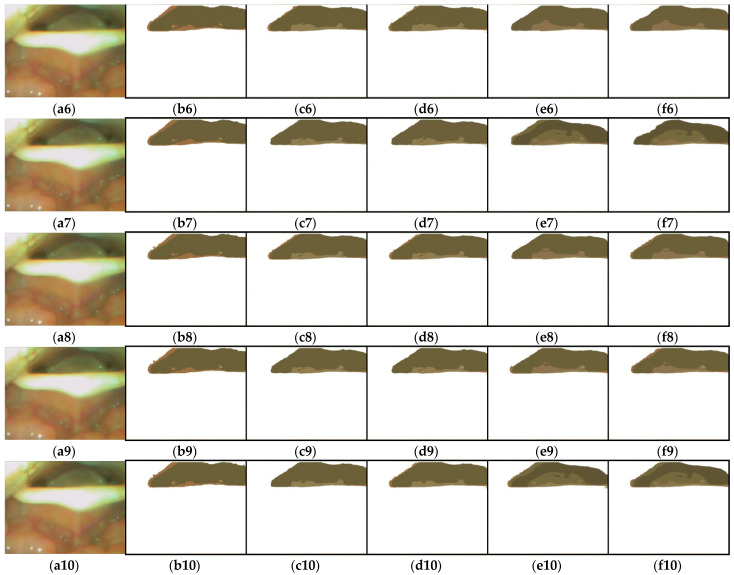
The AE-regions with the number of quantified colors (q=4,6,8,10,12): (**a1**–**a10**) t1–t10; (**b1**–**b10**) q=4; (**c1**–**c10**) q=6; (**d1**–**d10**) q=8; (**e1**–**e10**) q=10; (**f1**–**f10**) q=12.

**Figure 15:**
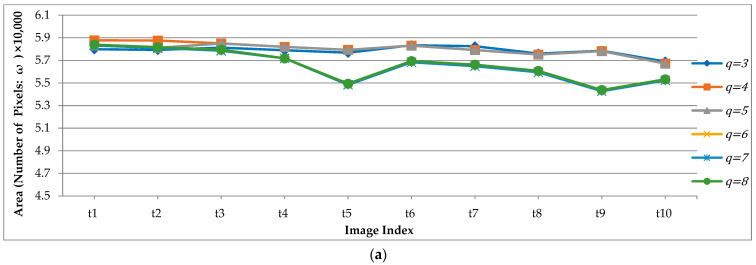

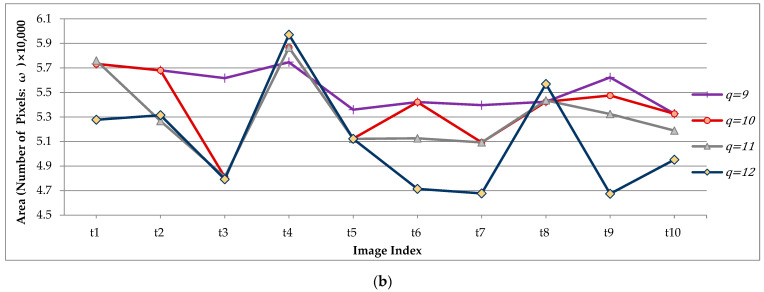
The areas of AE-region (ω) with the number of quantified colors: (**a**) q=3,4,5,6,7,and 8, and (**b**) q=9,10,11,and 12.

The AE-region detection method that we proposed is mainly based on setting a low number of quantized colors. The quantized color number of each experimental image was less than 12, and to understand the influence of quantized color number on AE-region detection, 10 continuous images (t1–t10, 1/3 s) were tested in this experiment for the AE-region, with the number of quantized colors ranging from 3 to 12. Figure 14 lists five AE-regions with the number of quantified colors (q=4,6,8,10,12). As shown in Figure 14, the greater the number of colors, the greater the number of $R_{j}$ in the AE-region, and the greater the change in the shape of the region. To obtain a better understanding of the change in the AE-regions, we quantified the number of AE-region pixels ($\omega_{n}=\left|A^{n}\right|$) for the number of colors (q ranging from 3 to 12) by 10, and the results are shown in Figure 15. To demonstrate subtle differences, we show q=3−8 in Figure 15a and q=9−12 in Figure 15b. Figure 15a shows that when q=6−8, the AE-region areas of the three color quantization numbers are closer, and when q=6−8, the areas are even closer, but the results are quite different. If the 10 continuous images were judged by humans, the AE-regions behind the epiglottis would gradually become smaller; thus, the number of colors during the detection of the AE-regions is more in line with the results of human observation.

### 3.5. Continuous Image AE-Region Change Analysis

**Figure 16:**
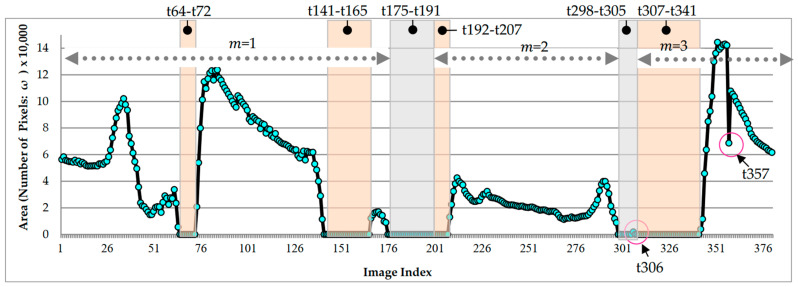
The AE-region areas ω in the 12.7 s video (total image number is 380).

**Figure 17:**
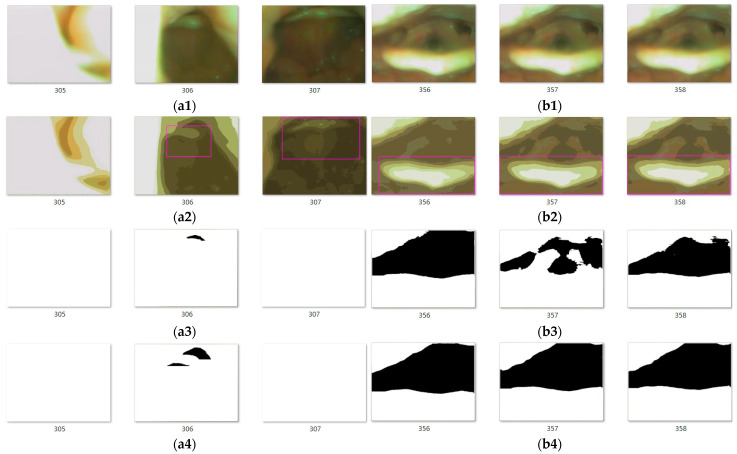
The AE-regions for images t306 and t357: (**a1**,**b1**) the original images; (**a2**,**b2**) the color-quantized images with the bounding box of epiglottis localization; (**a3**,**b3**) AE-regions (q=6); and (**a4**,**b4**) AE-regions (q=4).

We used a 12.7 s video (total image number was 380) recorded using the rhino-laryngoscope to perform the AE-region detection experiment. The color quantization number was set to q=6 (Figure 16). These 380 images include partial obstruction, complete obstruction, and rhino-laryngoscope sliding. The movement of the rhino-laryngoscope was used as the basis for updating the period number. When the epiglottis could not be detected, the rhino-laryngoscope was considered to be slipping. The two periods of t175–t191 and t298–t305 in the 380 images were regarded as the movement of the rhino-laryngoscope. Therefore, three periods (m=1,2,3) were detected in the 380 images; among them, the period m=1 is t1–t174, the period m=2 is t192–t29, and the period m=3 is t307–t341. The fully blocked part contains images t64–t72 (for 0.30 s), t141–t165 (for 0.83 s), and t307–t341 (for 1.16 s), while the rest are partially blocked and unblocked. During the analysis process, abnormal numerical values of the AE-region in images t306 and t357 (Figure 17(a1,b1), respectively) were detected. The color-quantized images are shown in Figure 17(a2,b2). The detection results of the AE-region in images t306 and t357 (q=6) are shown in Figure 17(a3,b3), respectively. In addition, the detection results of the color quantization number (q=4) are shown in Figure 17(a4,b4). The differences among Figure 17(b3,b4) are explained in Section 4.

**Figure 18:**
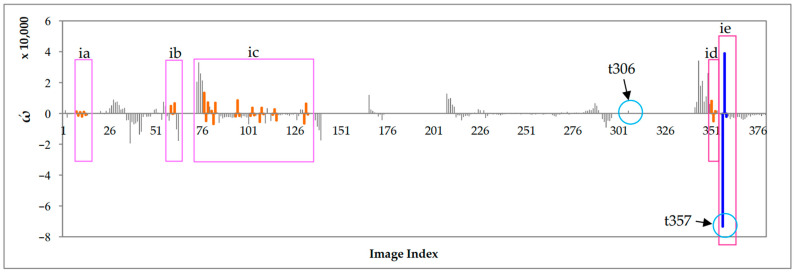
The backward difference ($\omega^{\prime }$) of the AE-region areas ω.

Among the images, the t306 image was detected to have an AE-region; however, the before and after images of t305 and t307 do not detect the epiglottis nor complete obstruction. Thus, we consider t306 to be abnormal data based on the results of the before and after images. In addition, there is a large difference between the detection results of images t357, t356, and t358 (Figure 17(b3)). We used inverse difference calculation ($\omega_{n+1}^{\prime }=\omega_{n+1}-\omega_{n}$) to obtain the results ($\omega^{\prime }$), which are shown in Figure 18, and examined the $\omega^{\prime }$ dataset for “positive and negative positive” and “negative positive and negative” values (as shown in Figure 18 in boxes ia–ie). If the difference value is higher than the threshold ($\left|\varepsilon \right|>30,000$, where the symbol $\left|.\right|$ is the absolute value operation), then it is regarded as abnormal. Therefore, the AE-region data of t357 were judged as abnormal, and we did not perform obstruction ratio calculations. Moreover, the computation time for the 380 images was 269.8 s, with an average computation time of 0.71 s per image.

### 3.6. Comparing the Proposed Method with the Clinical Judgment Results

This experiment used the same 380 images as the judgment targets, compared the results of the proposed method with the results obtained based on clinical judgment, and evaluated the appropriateness and practicability of our method through the comparison. During the comparison process, the period of tonsil interference was artificially determined. The mark was not included in the calculation of the obstruction ratio. The images of these two periods were t37–t62 and t156–t306. This experiment compares results based on current clinical diagnostic obstruction degrees, which were 0–49%, 50–75%, and 76–100%. The following are the results of the clinical judgment for the 380 continuous images (the clinical judgment was determined by the Chief of the Department of Otorhinolaryngology-Head and Neck Surgery at Kaohsiung Veterans General Hospital):

- In t1–t36, the epiglottis shrinks at least 50%;
- In t37–t62, there is tonsil interference (not included in the calculation of obstruction ratio);
- In t63–t73, there is no posterior pharyngeal wall in the image, but the epiglottic cartilage is sucked to the posterior pharyngeal wall, which is clinically judged to be 76–100% obstructed;
- In t74–t137, the epiglottis shrinks less than 50%;
- In t138–t155, there is no posterior pharyngeal wall in the image, but the epiglottic cartilage is sucked to the posterior pharyngeal wall, which is clinically judged to be 76–100% obstructed;
- In t156–t306, there is no posterior pharyngeal wall in the image, but there is tonsil interference (not included in the calculation of obstruction ratio);
- In t307–t343, the epiglottis cartilage is sucked to the direction of the posterior pharyngeal wall, which is clinically judged to be 76–100% obstructed;
- In t345–t380, the epiglottis shrinks less than 50%.

**Figure 19:**
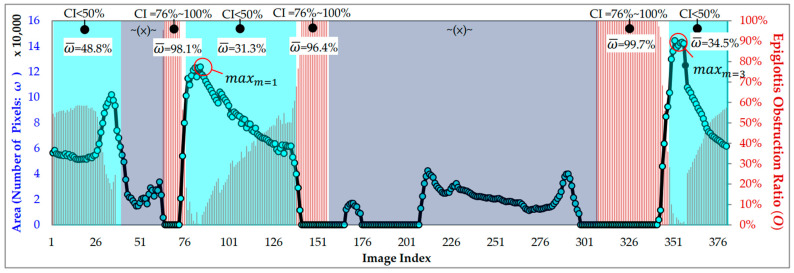
The results of epiglottis obstruction ratio (O).

There were three periods detected by our method (m=1,2,3). The two periods disturbed by the tonsils (t37–t62 and t156–t306) were excluded, and period 2 (t192–t297) fell within the period of tonsil interference, so we only counted periods 1 and 3. We estimated the maximum value of the AE-region ($\max\left\{{|A}_{m}^{n}|\right\}$) and calculated the obstruction ratio $O_{m}^{n}$ according to Equation (1), and the results are shown in Figure 19 (comparison of epiglottis obstruction ratio results). CI denotes the clinical judgment result, $\bar{\omega }$ is the average obstruction ratio in each period, the right vertical axis shows the epiglottis obstruction ratio (O), and the left vertical axis shows the number of pixels in the AE-region. The reddish-brown color histogram ($O_{m}^{n}$) is the epiglottis obstruction ratio of the image. We averaged the obstruction ratios of periods m=1 and m=3 to obtain the average value $\bar{\omega }$. The results are as follows:

- m=1 (t1–t36): $\bar{\omega }$ is 48.8% (clinical judgment CI = 0–49%).
- m=1 (t63–t73): $\bar{\omega }$ is 98.1% (clinical judgment CI = 76–100%);
- m=1 (t74–t137): $\bar{\omega }$ is 31.3% (clinical judgment CI = 0–49%);
- m=1 (t138–t155): $\bar{\omega }$ is 96.4% (clinical judgment CI = 76–100%);
- m=3 (t307–t343): $\bar{\omega }$ is 99.7% (clinical judgment CI = 76–100%);
- m=3 (t345–t380): $\bar{\omega }$ is 34.5% (clinical judgment CI = 0–49%).

After excluding the two periods of tonsil interference (t37–t62 and t156–t306), the average values obtained for the two periods are consistent with the results of the clinical judgment.

## 4. Discussion

### 4.1. Method Limitations

The proposed image processing method of the system is based on the YOLOv4 for the identification of the epiglottis and the low color quantization number of regions. Together with our proposed RPA to obtain the AE-region, the AE-region area data are plotted as a curve, and abnormal data are filtered out by comparing the image data of the front and back images with the threshold value without calculating the obstruction ratio. The quantitative data obtained by the proposed method can provide surgeons during diagnosis with the severity of obstruction of the epiglottis of patients, solve the problem that surgeons can only roughly judge the severity of obstruction, and help surgeons improve the accuracy of the diagnosis. In addition, surgeons can also explain to patients their situation with continuous data. Patients can better understand the time and trend of airway obstruction in their epiglottis. We use color quantization to convert the image into color regions. The distribution of color regions affects the result of merging AE-regions. A more complete region can be obtained with low color quantization, but larger meandering regions are also prone to appear. Unnecessary regions might be merged into the AE-region. Still, the AE-region changes between consecutive images are relatively gentle and the trend of continuous data changes is closer to the surgeon identification (Figure 15a). Regions with a higher color quantization number are smaller, fragmented, incomplete, and require more calculation time. The AE-region between consecutive images varies greatly, which is quite different from the surgeon identification (Figure 15b, q=12). Take Figure 17(a4,b4) as examples, if q=4, although t306 is also an abnormal value, t357 is a normal value. After comparing related experiments, we think that merging AE-regions with a low color quantization number is a better choice (q=4−8), so in the whole experiment, we take the middle-value q=6 as the color quantization number. In addition, we find the abnormal data by comparing the AE-region data of the before and after images with the threshold. We do not calculate the obstruction ratio from the abnormal data. From the experimental results, it can be observed that the results of our proposed method are consistent with the clinical judgment, which verifies the appropriateness and the practicability of this research method. 

### 4.2. Work Limitations

This study introduces a novel method of epiglottic obstruction image analysis to calculate the obstruction ratio. The calculation of the obstruction ratio has been expanded from three degrees to a numerical ratio. Compared to obstruction degrees, the obstruction ratio can provide surgeons with more accurate diagnoses, allowing patients to receive more suitable treatment options. However, the method proposed in this study and the experiments conducted still have several limitations, as follows:

1. The study specifically calculates the precise extent of airway obstruction in the epiglottic region only. The method designed for the study cannot calculate the obstruction ratio for other obstruction sites.
2. After calculating the AE-Region, a surgeon needs to determine the period affected by the tonsils, as this method cannot identify the tonsils.
3. This research did not conduct a control group analysis, meaning the study did not analyze the images of the epiglottic region in healthy individuals who do not have OSA. However, theoretically, the research method is equally applicable.

## 5. Conclusions

Surgeons determine the type of treatment a patient needs based on the severity of epiglottic obstruction. Traditional manual detection cannot accurately calculate the extent and duration of epiglottic obstruction, and previous automated methods for scoring epiglottic obstruction have primarily identified only three obstruction degrees. Moreover, these methods are better suited for distinguishing between obstructed and non-obstructed states. However, the accuracy of the three-level obstruction identification is insufficient, and its correlation with the apnea–hypopnea index (AHI) is not direct. The obstruction ratio provides a more precise diagnosis for surgeons. To accurately calculate the obstruction ratio, this study developed a system for calculating the epiglottic obstruction ratio. This system can perform in-depth image analysis of DISE videos and precisely calculate the epiglottis obstruction rate and duration. The system can assist surgeons in obtaining an accurate epiglottis obstruction ratio, enabling them to devise the best treatment plan for patients. This system is presented in a web format, offering cross-platform accessibility. Furthermore, the image analysis method proposed in this study integrates the YOLOv4, RPA, and other essential image processing algorithms. The proposed method only identifies a single epiglottic cartilage object. The YOLOv4 model’s training demonstrated a high mAP performance, with an overall high recognition rate (IoU threshold: 0.75 and F1 score: 91%). During the experiment of merging the EP-regions and AE-regions, it was also validated that the RPA could accurately merge the laryngeal cartilage region and the airway region. Additionally, this study explored the rationality of the number of color quantization areas. The last experiment utilized continuous data to compare the system’s calculated results with the clinical diagnostic outcomes determined by a surgeon. The system developed in this study could calculate the airway collapse’s percentage accuracy to 0.1%, and the average calculation time for a single image was only 0.71 s. Through experimental validation, the efficacy and practical value of this system’s image analysis method were proven. In future research, we will continue to develop image analysis methods to calculate the obstruction rate for the velum, oropharynx, and tongue areas, incorporate AHI index-related experiments, and enhance the functionality of this system.

## Data Availability

Not applicable.
